# Do Abnormal Serum Lipid Levels Increase the Risk of Chronic Low Back Pain? The Nord-Trøndelag Health Study

**DOI:** 10.1371/journal.pone.0108227

**Published:** 2014-09-18

**Authors:** Ingrid Heuch, Ivar Heuch, Knut Hagen, John-Anker Zwart

**Affiliations:** 1 Department of Neurology and FORMI, Oslo University Hospital, Oslo, Norway; 2 Department of Mathematics, University of Bergen, Bergen, Norway; 3 Department of Neuroscience, Norwegian University of Science and Technology, and Norwegian National Headache Centre, Department of Neurology, St. Olavs Hospital, Trondheim, Norway; 4 Faculty of Medicine, University of Oslo, Oslo, Norway; The James Cook University Hospital, United Kingdom

## Abstract

**Background:**

Cross-sectional studies suggest associations between abnormal lipid levels and prevalence of low back pain (LBP), but it is not known if there is any causal relationship.

**Objective:**

The objective was to determine, in a population-based prospective cohort study, whether there is any relation between levels of total cholesterol, high density lipoprotein (HDL) cholesterol and triglycerides and the probability of experiencing subsequent chronic (LBP), both among individuals with and without LBP at baseline.

**Methods:**

Information was collected in the community-based HUNT 2 (1995–1997) and HUNT 3 (2006–2008) surveys of an entire Norwegian county. Participants were 10,151 women and 8731 men aged 30–69 years, not affected by chronic LBP at baseline, and 3902 women and 2666 men with LBP at baseline. Eleven years later the participants indicated whether they currently suffered from chronic LBP.

**Results:**

Among women without LBP at baseline, HDL cholesterol levels were inversely associated and triglyceride levels positively associated with the risk of chronic LBP at end of follow-up in analyses adjusted for age only. Adjustment for the baseline factors education, work status, physical activity, smoking, blood pressure and in particular BMI largely removed these associations (RR: 0.96, 95% CI: 0.85–1.07 per mmol/l of HDL cholesterol; RR: 1.16, 95% CI: 0.94–1.42 per unit of lg(triglycerides)). Total cholesterol levels showed no associations. In women with LBP at baseline and men without LBP at baseline weaker relationships were observed. In men with LBP at baseline, an inverse association with HDL cholesterol remained after complete adjustment (RR: 0.83, 95% CI: 0.72–0.95 per mmol/l).

**Conclusion:**

Crude associations between lipid levels and risk of subsequent LBP in individuals without current LBP are mainly caused by confounding with body mass. However, an association with low HDL levels may still remain in men who are already affected and possibly experience a higher pain intensity.

## Introduction

Low back pain (LBP) is a common disabling disorder, representing a substantial economic burden to society [1]. Only in about 15% of the patients suffering from LBP is it possible to give a precise underlying pathoanatomical diagnosis [2]. It is therefore important to clarify general theories about causal relationships.

It has been suggested that LBP may be related to lumbar artery disease, with atherosclerosis in the feeding arteries producing reduced blood supply and disc degeneration [3]. LBP has been found more frequently in individuals with missing or narrow lumbar or middle sacral arteries [4] or with calcification in the abdominal aorta [5], [6]. In this way, LBP can possibly be compared to chest pain arising on the basis of atherosclerosis or to intermittent claudication with leg pain caused by impaired blood flow [7].

If the risk of LBP is affected by lesions in the arteries, risk factors for atherosclerosis should also be related to LBP, but results of epidemiological studies are equivocal [8], [9]. Only few studies [8]–[15] have dealt with associations between LBP and abnormal serum lipid levels, representing established risk factors for atherosclerosis [16]. In particular, large prospective studies of potential relationships between lipid levels and risk of LBP are needed.

The purpose of the present study was to investigate associations between serum lipid levels and occurrence of LBP using a prospective design, considering data from two large health surveys, carried out in a Norwegian county 11 years apart. As the course of LBP is often recurrent, with patients moving between acute and more chronic stages [17], risk factors may also be associated with subsequent occurrence in those already experiencing LBP. Thus one section of this study dealt with risk of LBP in participants not suffering from the disorder at baseline, while another part dealt with subsequent occurrence in participants with LBP at baseline.

Previous work based on the same cohort has shown positive associations between body mass index (BMI) and risk of LBP in both women and men [18]. High values of systolic and pulse pressure were also found to be related to a lower risk of LBP among women [19]. The present work includes adjustment for BMI and blood pressure, in addition to other potential confounders.

## Materials and Methods

### Participants

From 1995 to 1997, a large health survey, HUNT 2, was carried out in Nord-Trøndelag County in Norway. The entire population aged 20 years or more received a health questionnaire in which they indicated whether they had experienced LBP lasting for at least three months consecutively during the last year, which was regarded as chronic LBP. The participants underwent a clinical examination, including blood samples, with measurement of serum lipid levels [20]. In the HUNT 3 survey, carried out in 2006 to 2008 in the same county with a corresponding target population, similar questionnaires were distributed [21].

The present follow-up study was based on individual background information from HUNT 2 linked to information about chronic LBP collected in the subsequent HUNT 3 survey. The study aimed at the cohort consisting of 44,923 individuals who were 30 to 69 years old when they participated in the HUNT 2 survey and had information available on total cholesterol, high density lipoprotein (HDL) cholesterol, triglycerides and presence or absence of chronic LBP. Participants outside this age interval in HUNT 2 were excluded due to relatively low participation rates in the subsequent HUNT 3 survey [22]. During the period of follow-up, from HUNT 2 to HUNT 3, 2663 persons in this cohort died, 1686 persons left the county of Nord-Trøndelag, and one person disappeared. Furthermore, 15,123 members of the cohort residing in Nord-Trøndelag at the time of HUNT 3 did not participate or did not supply information about LBP. Thus a total of 25,450 persons, 14,053 women and 11,397 men, were available for analysis after follow-up, representing 62.7% of the remaining individuals resident in the county and 56.7% of the original cohort.

### Definitions

Participants in HUNT 2 had levels of total cholesterol, HDL cholesterol and triglycerides measured in non-fasting blood. Total cholesterol was measured applying an enzymatic colorimetric cholesterolesterase method, and HDL cholesterol was measured after precipitation with phosphortungsten and magnesium ions [20]. Triglycerides were also measured by an enzymatic colorimetric method. All lipid levels were categorized in five groups by quintiles, determined in the overall HUNT 2 population [13].

Systolic blood pressure was categorized in intervals less than 120, 120–139, 140–159 and 160 mm Hg or more, and diastolic blood pressure in intervals less than 80, 80–89, 90–99 and 100 mm Hg or more. BMI, defined as weight/height^2^, was subdivided into three groups, less than 25, 25–29.9, and 30 kg/m^2^ or more.

For work status, one category comprised those who were employed or carried out professional work. This category was further subdivided according to the level of physical activity at work, in four subcategories. The second category of work status included individuals temporarily unemployed, students and those in military service. The third category included pensioners and people receiving social security support, and the fourth category included women occupied full time with housework.

Leisure-time physical activity was categorized in 3 groups, as light activity only or hard activity less than 1 hour per week, hard activity 1–2 hours per week, and hard activity 3 hours per week or more. Hard activity was defined as activity leading to participants sweating or being out of breath. Physical activity in leisure time included moving to and from work.

Duration of education was considered in 3 groups, 9 years or less, 10–12 years, and 13 years or more. Categories of cigarette smoking represented current daily smoking, previous daily smoking, and never daily smoking. Age was categorized in 10-year intervals within the range 30–69 years.

### Statistical analyses

The percentage of chronic LBP at end of follow-up was computed within categories of total cholesterol, HDL cholesterol and triglycerides, separately for those without and with chronic LBP at baseline, to assess crude associations with lipid levels.

Associations adjusted for potential confounders were evaluated by generalized linear modeling for binomially distributed data with a log link. This procedure produced estimates of the risk ratio for any particular category of lipid levels relative to a reference category defined as the lowest category considered. As LBP is a relatively common disorder, this approach was preferred to logistic regression producing estimates of odds ratios which are poor approximations to risk ratios. In addition to categorical analyses involving quintiles of lipid levels, linear analyses were conducted with lipid levels considered as continuous variables. All such analyses were based on the actual recorded values of the lipid levels, not the quintiles. Likelihood tests were performed to test for trend. Triglyceride levels showed strongly skewed distributions and were logarithmically transformed (with base 10) before analysis.

One set of analyses included adjustment for age only. Further analyses were adjusted additionally for other factors potentially associated both with LBP and lipid levels, as education [23], [24], work status [25], [26], physical activity [27], [28], cigarette smoking [29], [30], BMI [18], [31] and systolic and diastolic blood pressure [19], [32]. All such factors were entered as categorical variables in the analyses. Because lipid measurements were performed on non-fasting blood, adjustment was made also for time between last meal and blood sampling. The main statistical strategy included tests for interaction between lipid levels and all variables adjusted for in the situations when a significant association was observed with a lipid level after full adjustment. In these tests, lipid levels were considered as continuous variables. Additional separate checks were made of the adequacy of the statistical model (Appendix S1), including tests for linearity in the effects of lipid levels and interaction terms for lipids showing no significant main effect.

Information was missing on some confounders in a minor fraction of the data set, and analyses with complete adjustment were based on a somewhat lower number of individuals than the basic age-adjusted analyses. All statistical analyses were carried out using IBM SPSS version 19 (IBM Corp, Armonk, NY).

### Ethics

Each participant in the HUNT 2 and HUNT 3 surveys signed a written informed consent regarding the collection and use of data for research purposes. This procedure was approved by the Norwegian Data Inspectorate and by the Regional Committee for Ethics in Medical Research. The analysis was approved by the Regional Committee for Ethics in Medical Research.

## Results

### Associations among participants without LBP at baseline

A total of 10,151 women and 8731 men reported not having chronic LBP at baseline. In this group, 2028 women (20.0%) and 1226 men (14.0%) reported chronic LBP at end of follow-up. No definite association was seen between crude risk of chronic LBP and total cholesterol in either sex among the participants free of LBP at baseline (Table 1), but moderate inverse associations were suggested with levels of HDL in both women and men. The crude risk of LBP showed a weak tendency to increase with increasing levels of triglycerides among these participants.

**Table 1:** Proportion of individuals with chronic LBP at end of follow-up, by lipid levels and LBP status at baseline.

	Among individuals without LBP at baseline				Among individuals with LBP at Baseline			
	Women		Men		Women		Men	
	Total	With LBP at end offollow-up (%)	Total	With LBP at end offollow-up (%)	Total	With LBP at end offollow-up (%)	Total	With LBP at end offollow-up (%)
**Total cholesterol (mmol/l)**								
≤4.8	2173	442 (20.3)	1421	200 (14.1)	643	341 (53.0)	400	192 (48.0)
4.9–5.5	2316	441 (19.0)	2015	288 (14.3)	791	452 (57.1)	572	269 (47.0)
5.6–6.1	1957	419 (21.4)	1947	286 (14.7)	788	507 (64.3)	595	287 (48.2)
6.2–6.9	1873	364 (19.4)	1932	236 (12.2)	836	502 (60.0)	601	296 (49.3)
≥7	1832	362 (19.8)	1416	216 (15.3)	844	525 (62.2)	498	226 (45.4)
**HDL cholesterol (mmol/l)**								
≤1.0	847	190 (22.4)	2520	389 (15.4)	410	265 (64.6)	806	420 (52.1)
1.1–1.2	1497	340 (22.7)	2300	315 (13.7)	636	383 (60.2)	698	328 (47.0)
1.3–1.4	2169	442 (20.4)	1856	249 (13.4)	863	528 (61.2)	519	239 (46.1)
1.5–1.7	2966	542 (18.3)	1447	198 (13.7)	1113	631 (56.7)	436	188 (43.1)
≥1.8	2672	514 (19.2)	608	75 (12.3)	880	520 (59.1)	207	95 (45.9)
**Triglycerides (mmol/l)**								
≤0.94	3155	599 (19.0)	1129	137 (12.1)	969	533 (55.0)	336	152 (45.2)
0.95–1.29	2481	466 (18.8)	1541	214 (13.9)	845	510 (60.4)	470	220 (46.8)
1.30–1.70	1872	393 (21.0)	1768	244 (13.8)	797	476 (59.7)	529	253 (47.8)
1.71–2.38	1537	346 (22.5)	2008	267 (13.3)	712	422 (59.3)	595	262 (44.0)
≥2.38	1106	224 (20.3)	2285	364 (15.9)	579	386 (66.7)	736	383 (52.0)

LBP, low back pain.

Generalized linear modeling revealed no associations with total cholesterol (Table 2). Analyses adjusted for age only showed inverse associations with HDL levels and positive associations with triglyceride levels, although statistical significance was not reached for HDL in men (Table 2). However, after complete adjustment for other potential risk factors, these associations were substantially weakened and were no longer significant. Separate analyses carried out on participants with known values for all factors adjusted for, but with age adjustment only, gave very similar risk estimates to the age adjusted values shown in Table 2.

**Table 2:** Associations between lipid levels and risk of chronic LBP among individuals without chronic LBP at baseline.

	Women		Men	
	Adjustment for age only	Complete adjustment*	Adjustment for age only	Complete adjustment*
	Risk ratio (95% CI)	Risk ratio (95% CI)	Risk ratio (95% CI)	Risk ratio (95% CI)
**Number of individuals**	10151	9159	8731	8078
**Total cholesterol (mmol/l)**				
≤4.8	1.00 (reference)	1.00 (reference)	1.00 (reference)	1.00 (reference)
4.9–5.5	0.93 (0.82–1.05)	0.90 (0.79–1.02)	1.02 (0.86–1.21)	0.99 (0.83–1.18)
5.6–6.1	1.04 (0.92–1.18)	1.04 (0.91–1.18)	1.06 (0.89–1.25)	1.00 (0.84–1.19)
6.2–6.9	0.95 (0.83–1.08)	0.91 (0.79–1.05)	0.88 (0.74–1.06)	0.83 (0.69–1.00)
≥7	0.97 (0.84–1.12)	0.93 (0.80–1.08)	1.11 (0.92–1.33)	1.00 (0.83–1.21)
Continuous results				
Per mmol/l	0.99 (0.95–1.02)	0.98 (0.94–1.02)	1.00 (0.96–1.06)	0.97 (0.92–1.02)
* P* for log-linear association	0.46	0.27	0.87	0.29
**HDL cholesterol (mmol/l)**				
≤1.0	1.00 (reference)	1.00 (reference)	1.00 (reference)	1.00 (reference)
1.1–1.2	1.02 (0.87–1.18)	1.05 (0.88–1.24)	0.89 (0.78–1.02)	0.96 (0.83–1.11)
1.3–1.4	0.91 (0.78–1.06)	0.98 (0.83–1.15)	0.88 (0.76–1.01)	0.95 (0.82–1.11)
1.5–1.7	0.81 (0.70–0.94)	0.89 (0.76–1.04)	0.90 (0.77–1.05)	0.94 (0.79–1.11)
≥1.8	0.86 (0.74–0.99)	0.96 (0.82–1.13)	0.81 (0.64–1.02)	0.86 (0.67–1.11)
Continuous results				
Per mmol/l	0.87 (0.78–0.97)	0.96 (0.85–1.07)	0.86 (0.73–1.01)	0.91 (0.77–1.08)
* P* for log-linear association	0.008	0.45	0.07	0.29
**Triglycerides (mmol/l)**				
≤0.94	1.00 (reference)	1.00 (reference)	1.00 (reference)	1.00 (reference)
0.95–1.29	1.00 (0.89–1.11)	0.95 (0.85–1.07)	1.15 (0.94–1.40)	1.09 (0.88–1.34)
1.30–1.70	1.12 (0.99–1.25)	1.06 (0.93–1.20)	1.15 (0.94–1.40)	1.10 (0.90–1.36)
1.71–2.38	1.20 (1.07–1.35)	1.12 (0.99–1.28)	1.10 (0.91–1.34)	1.05 (0.85–1.29)
≥2.38	1.08 (0.94–1.25)	0.99 (0.84–1.16)	1.31 (1.09–1.58)	1.20 (0.98–1.47)
Continuous results				
Per unit of lg(triglycerides)	1.32 (1.10–1.58)	1.16 (0.94–1.42)	1.40 (1.12–1.74)	1.24 (0.96–1.58)
* P* for log-linear association	0.003	0.18	0.003	0.10

LBP, low back pain; CI confidence interval.

*Adjustment for age, education, work status, physical activity, smoking, BMI, blood pressure and time between last meal and blood sampling.

To determine which factor contributed most to the effect of the adjustment, analyses were also carried out with adjustment for each separate factor in addition to age. Adjustment for education, smoking, physical activity, work status and blood pressure had relatively little influence on associations between triglyceride levels and chronic LBP (Table 3), whereas adjustment for BMI led to substantial weakening of the associations. A similar tendency was seen for associations with HDL levels (Table 3), except that adjustment for smoking had an effect on the association in women of about the same magnitude as that for BMI. As total cholesterol levels showed no associations with LBP, neither with adjustment for age nor with complete adjustment, results are not shown for this variable in Table 3. No significant interaction was observed between lipid levels and BMI.

**Table 3:** Log-linear associations between HDL and triglyceride levels and risk of chronic LBP among individuals without chronic LBP at baseline, with adjustment for different variables.

	Women		Men	
	Risk ratio per mmol/l ofHDL cholesterol*(95% CI)	Risk ratio per unit oflg(triglycerides)*(95% CI)	Risk ratio per mmol/l ofHDL cholesterol*(95% CI)	Risk ratio per unit oflg(triglycerides)*(95% CI)
**Adjustment for age only**	0.87 (0.78–0.97)	1.32 (1.10–1.58)	0.86 (0.73–1.01)	1.40 (1.12–1.74)
**Additional adjustment** †				
Education	0.88 (0.79–0.98)	1.27 (1.06–1.52)	0.86 (0.73–1.01)	1.34 (1.08–1.68)
Smoking	0.91 (0.82–1.01)	1.28 (1.07–1.54)	0.88 (0.75–1.03)	1.36 (1.09–1.70)
Leisure time physical activity	0.88 (0.79–0.97)	1.29 (1.07–1.55)	0.86 (0.73–1.02)	1.42 (1.13–1.77)
Work status, including physical activity at work	0.87 (0.78–0.97)	1.29 (1.08–1.55)	0.83 (0.71–0.98)	1.40 (1.12–1.75)
BMI	0.91 (0.82–1.01)	1.19 (0.99–1.45)	0.94 (0.80–1.11)	1.21 (0.95–1.52)
Blood pressure	0.87 (0.78–0.96)	1.37 (1.14–1.64)	0.87 (0.74–1.03)	1.37 (1.09–1.71)
**Complete adjustment** ‡	0.96 (0.85–1.07)	1.16 (0.94–1.42)	0.91 (0.77–1.08)	1.24 (0.96–1.58)

LBP, low back pain; CI confidence interval.

*Considered as a continuous variable.

† Adjustment for age and factor indicated in each case.

‡ Adjustment for age, education, work status, physical activity, smoking, BMI, blood pressure and time between last meal and blood sampling.

### Associations among participants with LBP at baseline

In the group who reported chronic LBP at baseline, including 3902 women and 2666 men, a total of 2327 women (59.6%) and 1270 men (47.6%) also experienced chronic LBP at end of follow-up. Crude percentages did not indicate any relation between occurrence of chronic LBP in this group and total cholesterol (Table 1), while only weak inverse associations with HDL and positive associations with triglycerides were suggested.

Associations indicated with triglyceride levels largely disappeared after complete adjustment for other risk factors (Table 4). This was also the case with the inverse association with HDL levels in women, but in men the association persisted after complete adjustment. Separate risk ratios relative to levels ≤1.0 mmol/l were 0.92 (95% confidence interval [CI] 0.83–1.02), 0.90 (95% CI 0.80–1.01), 0.85 (95% CI 0.74–0.97) and 0.89 (95% CI 0.75–1.07) for HDL levels in the 1.1–1.2, 1.3–1.4, 1.5–1.7 and ≥1.8 mmol/l intervals, respectively. No significant interaction was found between levels of HDL in this case and age or any other variable adjusted for.

**Table 4:** Log-linear associations between lipid levels and probability of chronic LBP among individuals with chronic LBP at baseline.

	Women		Men	
	Adjustment for age only	Complete adjustment*	Adjustment for age only	Complete adjustment*
	Risk ratio (95% CI)	Risk ratio (95% CI)	Risk ratio (95% CI)	Risk ratio (95% CI)
**Number of individuals**	3902	3418	2666	2414
**Total cholesterol**				
Per mmol/l†	1.02 (0.99–1.04)	1.00 (0.98–1.03)	0.99 (0.96–1.03)	0.98 (0.94–1.01)
* P* †	0.20	0.93	0.66	0.19
**HDL cholesterol**				
Per mmol/l†	0.94 (0.88–1.00)	0.99 (0.92–1.07)	0.84 (0.74–0.96)	0.83 (0.72–0.95)
* P* †	0.06	0.81	0.007	0.007
**Triglycerides**				
Per unit of lg(triglycerides)†	1.24 (1.10–1.40)	1.08 (0.94–1.24)	1.17 (0.99–1.38)	1.07 (0.88–1.29)
* P* †	<0.001	0.27	0.07	0.51

LBP, low back pain; CI confidence interval.

*Adjustment for age, education, work status, physical activity, smoking, BMI, blood pressure and time between last meal and blood sampling.

† Lipid levels considered as continuous variables.

## Discussion

In initial age-adjusted analyses of this prospective data set, the risk of chronic LBP was inversely associated with HDL levels and positively associated with triglyceride levels. However, further adjustment indicated that these associations were, at least to some extent, a product of confounding by other risk factors, in particular BMI. Yet in men who were already affected by chronic LBP at baseline, the inverse association between HDL and subsequent occurrence of chronic LBP remained after complete adjustment.

An important strength of this study was the opportunity to take into account relevant potential confounders, although information on such factors was missing in 8.7% of the subjects without LBP at baseline and 11.2% of those with LBP at that time. However, as the results with age adjustment only were very similar regardless of whether these subjects were included or not, missing values probably had little influence on the estimates concerned. Laboratory measurements were carried out by standardized procedures and the period between assessment of risk factors and final reporting of back pain was relatively long. Yet information on LBP was only available at baseline and end of follow-up, and other changes in back pain status during the intervening period were not recorded. Moreover, information on LBP was based on self-reported data and did not rely on a specific clinical examination.

Unfortunately no information was available on pain intensity or on cholesterol lowering medication. If abnormal lipid levels form part of a causal pathway to LBP, it is not obvious that use of such medication constitutes a potential confounder, but with sufficient information it might have been reasonable to analyse data separately for users and non-users. Furthermore, lipid measurements made on non-fasting blood may not represent the correct average over time for each participant. An attempt was made to compensate to some extent for this problem by adjusting for time since last meal. Finally, despite a relatively high response rate in the first survey, the response was lower at the second survey. There is no particular reason, however, why this should have introduced a noticable bias in the risk estimates.

Many patients who are affected by back pain recover after a certain period but are later prone to recurrent episodes [33]. In our study, chronic LBP was defined in the conventional manner requiring a continuous duration of at least 3 months [34], but if additional information had been available on pain intensity over an extended period, a more precise definition [35] could have made it easier to select those who were genuinely suffering from long-lasting LBP. However, even with a stricter definition of chronic LBP, some patients will later recover [35], so the percentage of chronic LBP observed at follow-up in our data among those with LBP at baseline is not surprising. Under these conditions it is not easy to distinguish between risk factors among subjects without LBP and factors affecting recurrence or persistence in those already experiencing LBP. This was the motivation in our study for considering assocations with lipid levels also among subjects with LBP at baseline.

To maintain the temporal relation between potential risk factors and the outcome variable representing LBP at follow-up, no other individual information collected at follow-up was included as predictors in our analyses. It is thus unlikely that the final disease status has influenced the values recorded for risk factors. Previous prospective epidemiological studies of relationships between lipid levels and occurrence of back pain have been based on follow-up of occupational cohorts. A long-term Finnish study of employees in an engineering company [9], [36] showed positive associations with tryglyceride levels for both local and radiating LBP, and associations persisted after adjustment for BMI. Results for total cholesterol were more ambiguous. A prospective study of British civil servants [8] revealed a positive relationship between sick-leave due to back pain and triglyceride levels, and the relation was essentially retained after adjustment for BMI and other relevant factors. No clear association emerged with total cholesterol. Finally, an American study of a cohort involved in petroleum-manufacturing [14] produced a positive assocation with triglycerides which largely disappeared after adjustment for obesity and other risk factors.

A cross-sectional study of the HUNT 2 population [13] showed an inverse association with HDL and a positive association with triglyceride levels, which was still significant in women after adjustment for BMI and other potential confounders. No definite associations were found between back pain and lipid levels in other cross-sectional studies [10]–[12], [15], but in many cases results were not adjusted for other risk factors. However, in a Finnish study [37] associations were observed in men between prevalence of sciatica and levels of total and low density lipoprotein (LDL) cholesterol and triglycerides but not HDL cholesterol, in analyses adjusted for BMI and other confounders. In our study, we did not consider the LDL level as a potential risk factor for LBP as no separate measurements of LDL were available, but to a large extent total cholesterol levels reflect LDL.

The notion that LBP may be related to lumbar artery disease [3] was partly based on a comparison of postmortem angiograms showing more missing or narrow lumbar or middle sacral arteries in subjects with LBP [4]. The hypothesis was supported by subsequent studies showing an association between LBP and occluded or narrowed lumbar arteries [38], [39] or presence of atherosclerotic calcifications [5], [6]. Calcification of the abdominal aorta has also been associated with intervertebral disc degeneration in several studies [5], [39]–[41]. The relationship with LBP is presumably mediated by reduced blood supply [3], [42], and an association has been indicated between lumbar arterial status and diffusion in the discs [43], [44]. A recent study, however, found an increased blood flow in the lumbar arteries in LBP patients [45]. Regarding lipid levels, one study found no major differences between patients with lumbar spinal stenosis and controls [46], although associations have been observed between levels of LDL cholesterol and disc degeneration [47] and total cholesterol and disc herniation [48]. It has also been suggested that statin use may retard the process of disc degeneration [49].

Thus although there are many indications that arterial status may be related to pain arising in the lumbar region, no firm link has been established between lipid levels, intermediate factors and back pain mechanisms. Abnormal levels of triglycerides and total and HDL cholesterol have been regarded as established independent risk factors for atherosclerosis and cardiovascular disease [50]. In view of the hypothesis that atherosclerosis may cause LBP, it may seem peculiar that no relation was indicated at all with total cholesterol in our data set. For stroke, however, total cholesterol levels do not seem to be associated with risk at the population level [50], in contrast to associations with HDL and triglycerides, so this is not a unique finding.

It is possible that lipid levels can influence the risk of LBP by other mechanisms. Thus dyslipidemia is related to inflammation [51], which may be linked to LBP in other ways [9]. Moreover, lipid levels may be associated with lumbar spine bone mineral density [52], which could play a role in the development of LBP.

Several other variables are associated with lipid levels at the population level and also constitute potential risk factors for LBP. BMI occupies a special position in this regard, as it is a relatively strong risk factor for LBP [18] and at the same time shows substantial associations with lipid levels over long periods of life [31]. In our study, adjustment for BMI had a major effect on the triglyceride and HDL associations with chronic LBP. Some other studies have shown similar effects of adjustment for BMI [13], [14], although this is not a consistent finding [8], [9]. If lipid levels influence BMI, it is possible that this to some extent represents an overadjustment, so that the true relations between risk of LBP and lipid levels are somewhere between those shown here with age adjustment only and complete adjustment.

The particular association with HDL remaining after complete adjustment, among men with chronic LBP at baseline, may represent a chance finding among many statistical tests. It is still reasonable that low HDL cholesterol levels may have an effect different from high triglyceride levels on predisposing factors for LBP. The contrast between men and women in this regard may reflect general sex differences for LBP, as the prevalence is higher in women and women may have a different pain threshold [53]. In the group of men who reported chronic LBP at baseline, subsequent LBP could represent a somewhat different, more permanent condition, possibly with higher pain intensity. Underlying associations with lipid levels may be more pronounced for particularly severe back pain, as suggested for LDL by one study [39]. General high-intensity chronic pain has also been found to be related to low HDL levels [54].

Unfortunately the present study does not provide any definite answer concerning associations between lipid levels and risk of LBP. If the LBP related to atherosclerosis of abdominal arteries represents a relatively small proportion of all LBP cases, a better classification of this heterogeneous medical condition may be important in future studies. Information on intensity and more precise duration of pain may be essential. A detailed medical classification may also help in delineating other specific causal mechanisms, although this may be difficult to achieve in large population-based studies.

## Supporting Information

Appendix S1 **Assessment of the statistical model.**
(PDF)
