# Supplementary material for: Do Abnormal Serum Lipid Levels Increase the Risk of Chronic Low Back Pain? The Nord-Trøndelag Health Study
Source: PLoS One. 2014 Sep 18;9(9):e108227. doi: 10.1371/journal.pone.0108227 (PMC4169450; doi:10.1371/journal.pone.0108227)
Supplement: Appendix S1 — Assessment of the statistical model. (PDF) [file pone.0108227.s001.pdf]

Appendix S1. Assessment of the statistical model

Model adequacy

To assess the adequacy of the statistical model used in evaluating associations between lipid levels and occurrence of low back pain (LBP), the data set was also analysed with various other sets of explanatory factors. Appendix Table 1 shows the results for risk of LBP at follow-up among the individuals without LBP at baseline. To make results comparable, all analyses were restricted to the 9159 women and 8078 men with complete information on all factors included in the analysis with complete adjustment.

For each model considered, the table displays the deviance, defined as twice the difference in log-likelihood between the corresponding saturated model and the model in question [1], and the corresponding degrees of freedom. Deviances were not computed as defined in SPSS, but rather considering each observation as a group on its own with a separate parameter estimated in the saturated model.

All models considered in Appendix Table 1 had deviances less than the relevant number of degrees of freedom. Each model can be compared to a simpler nested one by considering the difference in deviance as a chi-squared statistic $\chi^2(\nu)$ with the degrees of freedom ν given by the corresponding difference in the table. Thus the model with age only compared to the null model corresponded to $\chi^2(1) = 3.26$ for women and 9.27 for men. This gives some support to the notion that age is a factor that should be included in the model for men, although the support is much weaker for women.

Extending the model by incorporating all potential confounders considered as explanatory factors, a comparison with the model based on age only gave $\chi^2(21) = 92.35$ for women and $\chi^2(20) = 96.54$ for men. (The difference in degrees of freeedom is due to the different classification of occupation for women.) Both values definitely indicate that a better description is achieved by including the potential confounders.

For each separate lipid level, the model was then extended by including first a linear term, then an additional quadratic term and finally a cubic term, as shown in Appendix Table 1. Addition of linear terms corresponds to test results shown in Table 2 in the paper. For quadratic terms, only $\chi^2(1) = 2.83$ for HDL in women and $\chi^2(1) = 3.06$ for total cholesterol in men gave some weak support to non-linear relationships, but neither value was significant in the conventional sense. There was very little support of more complex relationships represented by cubic terms for any lipid level.

Analysis of the corresponding data for recurrence of LBP among the individuals with LBP at baseline produced a similar overall picture. In particular, no clear indications were found of non-linear relationships with lipid levels.

Interactions with effects of lipid levels

Models including linear effects of lipid levels, with complete adjustment, were explored with additional interaction terms between each lipid variable and the factors adjusted for. For risk of LBP among individuals without LBP at baseline, the following terms were statistically significant at the formal 0.05 level:

Among women, an interaction between the total cholesterol level and cigarette smoking was indicated ($\chi^2(2) = 7.73$), with a positive association between cholesterol and LBP being suggested

among former smokers. An interaction was also indicated between HDL cholesterol and systolic blood pressure (χ^2 (3) = 8.25), with a more pronounced inverse association among women with high blood pressure. Finally, an interaction was suggested in women between triglycerides and education (χ^2 (2) = 9.25), with a stronger positive association with LBP among women with a longer duration of education.

In men, the only interaction indicated for risk of LBP among those without LBP at baseline appeared between total cholesterol and work status (χ^2 (5) = 14.67). A more pronounced positive association with cholesterol level was observed among those not being employed at the time of interview.

Thus 4 interactions (or 7.4%) out of a total of 54 considered for individuals without LBP at baseline reached formal significance at the 0.05 level. For associations with recurrence of LBP among those with LBP at baseline, a total of 3 interactions (or 5.5%) showed similar statistical significance. The factors involved did not show any systematic pattern across lipid levels. Thus there seems to be no compelling reason to reject the main statistical models applied without interaction terms.

Goodness of fit

To test for goodness-of-fit, a version of the Hosmer-Lemeshow test based on deciles of predicted risks [2] was applied. This test was originally developed for logistic regression, but results of Blizzard and Hosmer [3] indicate that a corresponding procedure can also be used in connection with risks computed on the basis of a log-binomial regression model. The test is essentially performed by computing a chi-square statistic comparing the observed and expected numbers of individuals with LBP in the various deciles of predicted risk.

The test was applied to all 12 situations representing 3 lipid levels, women and men, and risk among those without LBP at baseline or recurrence in those with LBP at that time. Only models with complete adjustment for potential confounders were considered. At the significance level of 0.05, the test indicated deviations from the model in one situation only, for triglycerides and recurrence of LBP among women (P = 0.03). No systematic pattern of deviations between observed and expected number of individuals with LBP was seen in this situation or any other situation considered.

Discrimination

The model applied to analyse associations between lipid levels and occurrence of LBP was not intended to be used for classification of individuals. It may still be of interest to consider the discriminatory power of the model, in particular for different sets of variables adjusted for. Predicted values of the probability of LBP were used to generate a classification indicating whether an individual would experience LBP or not by setting a particular threshold value. In combination with the actual observed state at follow-up, these values could be used to estimate sensitivity and specificity. By considering all possible threshold values, this procedure generated a receiver operating curve (ROC), with the area under the curve (AUC) indicating how well the discrimination procedure worked [2]. Scores of AUC close to 0.5 represent a classification procedure of little value, with scores closer to 1.0 being optimal.

Appendix Table 2 presents the results with different sets of explanatory variables, separately for individuals with and without LBP at baseline and for women and men. Clearly the models based on age only provide poor discrimination. Models including all variables adjusted for lead to AUC scores

approaching 0.6. Adding a lipid level to the model as a continuous explanatory variable gives in most cases very little improvement, which is not surprising in view of the weak associations involved. Use of categorical variables representing lipid levels only led to marginal further improvement and these results are not included in the table. Among individuals without LBP at baseline, the discriminatory power seemed to be slightly better among men than women, but this difference was lost focusing on individuals with LBP at baseline.

Conclusion

Although there are some indications in particular cases that the main statistical models do not cover all relevant features of the data, it appears that there is no strong evidence against the kind of model applied. Assuming linearity in effects of lipid variables and restricting the principal analyses to main effects only, without interactions, seem to be justified. However, if the purpose had been to discriminate between individuals who will later experience LBP and those who will not, the models would not be satisfactory. This applies regardless of whether lipid levels are included as explanatory factors or not. This finding is not unique to the present study. Current models for the prognosis of back pain have in general poor predictive power [4].

Appendix References

1. McCullagh P, Nelder JA (1989) Generalized linear models. 2nd ed. London: Chapman & Hall.
2. Hosmer DW, Lemeshow S, Sturdivant RX (2013) Applied logistic regression. 3rd ed. Hoboken, NJ: Wiley.
3. Blizzard L, Hosmer DW (2006) Parameter estimation and goodness-of-fit in log binomial regression. Biom J 48: 5-22
4. Hayden JA, Dunn KM, van der Windt DA, Shaw WS (2010) What is the prognosis of back pain? Best Pract Res Clin Rheumatol 24: 167-179.

Appendix Table 1. Fit of generalized linear models used to predict the risk of LBP among individuals without LBP at baseline, with different sets of explanatory variables included, by sex.

	**Women**		**Men**	
**Effects in model**	**Deviance**	**Degrees of freedom**	**Deviance**	**Degrees of freedom**
Null model*	9100.00	9158	6531.98	8077
Age	9096.74	9155	6522.71	8074
All factors adjusted for†	9004.39	9134	6426.17	8054
Total cholesterol‡, and factors adjusted for	9003.16	9133	6425.05	8053
Total cholesterol, with square, and factors adjusted for	9003.16	9132	6421.99	8052
Total cholesterol, with square and cube, and factors adjusted for	9002.60	9131	6421.93	8051
HDL cholesterol‡, and factors adjusted for	9003.81	9133	6425.03	8053
HDL cholesterol, with square, and factors adjusted for	9000.98	9132	6424.98	8052
HDL cholesterol, with square and cube, and factors adjusted for	8999.05	9131	6424.78	8051
Triglycerides‡, and factors adjusted for	9002.58	9133	6423.41	8053
Triglycerides, with square, and factors adjusted for	8999.91	9132	6423.38	8052
Triglycerides, with square and cube, and factors adjusted for	8999.87	9131	6422.90	8051

LBP, low back pain.

*Includes intercept as only parameter.

†Age, education, work status, physical activity, smoking, BMI, blood pressure and time between last meal and blood sampling.

‡Lipid levels considered as continuous variables.

Appendix Table 2. Area under curve (AUC) for receiver operating curve (ROC) used to discriminate between individuals with and without future LBP, on the basis of models including different explanatory factors

	**Individuals without LBP at baseline**		**Individuals with LBP at baseline**	
**Effects in model**	**Women**	**Men**	**Women**	**Men**
Age	0.512	0.527	0.536	0.528
All factors adjusted for*	0.574	0.595	0.598	0.586
Total cholesterol†, and factors adjusted for	0.575	0.596	0.598	0.589
HDL cholesterol†, and factors adjusted for	0.574	0.595	0.598	0.592
Triglycerides†, and factors adjusted for	0.574	0.597	0.599	0.586

LBP, low back pain.

*Age, education, work status, physical activity, smoking, BMI, blood pressure and time between last meal and blood sampling.

†Lipid levels considered as continuous variables.
